# The Importance of Peripheral Nerves in Adipose Tissue for the Regulation of Energy Balance

**DOI:** 10.3390/biology8010010

**Published:** 2019-02-12

**Authors:** Magdalena Blaszkiewicz, Jake W. Willows, Cory P. Johnson, Kristy L. Townsend

**Affiliations:** 1 Graduate School of Biomedical Science and Engineering, University of Maine, Orono, ME 04469, USA; magdalena.blaszkiewicz@maine.edu (M.B.); cory.p.johnson@maine.edu (C.P.J.); 2 School of Biology and Ecology, University of Maine, Orono, ME 04469, USA; jake.willows@maine.edu

**Keywords:** adipose innervation, BAT, WAT, thermogenesis, sympathetic, brain–adipose communication, adipose peripheral nerves, adipose neuropathy, neural plasticity

## Abstract

Brown and white adipose tissues are essential for maintenance of proper energy balance and metabolic health. In order to function efficiently, these tissues require both endocrine and neural communication with the brain. Brown adipose tissue (BAT), as well as the inducible brown adipocytes that appear in white adipose tissue (WAT) after simulation, are thermogenic and energy expending. This uncoupling protein 1 (UCP1)-mediated process requires input from sympathetic nerves releasing norepinephrine. In addition to sympathetic noradrenergic signaling, adipose tissue contains sensory nerves that may be important for relaying fuel status to the brain. Chemical and surgical denervation studies of both WAT and BAT have clearly demonstrated the role of peripheral nerves in browning, thermogenesis, lipolysis, and adipogenesis. However, much is still unknown about which subtypes of nerves are present in BAT versus WAT, what nerve products are released from adipose nerves and how they act to mediate metabolic homeostasis, as well as which cell types in adipose are receiving synaptic input. Recent advances in whole-depot imaging and quantification of adipose nerve fibers, as well as other new research findings, have reinvigorated this field of research. This review summarizes the history of research into adipose innervation and brain–adipose communication, and also covers landmark and recent research on this topic to outline what we currently know and do not know about adipose tissue nerve supply and communication with the brain.

## 1. Introduction

The brain, and chiefly the hypothalamus, is responsible for coordinating energy balance by integrating signals coming from the periphery. These signals include important endocrine hormones and nutrients from the circulatory system, but also feedback from sensory nerves in peripheral tissues and organs. In turn, the brain can communicate outwardly to these tissues and organs through distinct efferent peripheral nerves, such as sympathetic nerves. For adipose tissue, this incoming sympathetic drive and resulting release of the neurotransmitter norepinephrine (NE) is critical for metabolic processes including lipolysis, adipogenesis, and browning. In recent years, several research groups (including ours) have reinvigorated the study of how neural innervation of adipose tissue is regulated, and many new discoveries and advancements in tissue imaging have been made. Lessons from non-adipose tissues, and also from other research animal models, have contributed important knowledge about how peripheral nerves are regulated, including by crosstalk with nearby immune cells that are capable of impacting neural plasticity. Adipose tissue innervation has been studied since the 1890s, and seminal studies were contributed by several research groups in the last few decades. In this review we summarize historic, landmark, and recent studies that provide insights into the roles of adipose tissue nerves in metabolic function. 

## 2. Innervation of Adipose Tissues

### 2.1. Innervation of Brown Adipose Tissue (BAT)

The notion that neural innervation exists in adipose tissues is not a new one; from an anatomical and functional perspective, all tissues of the body should be innervated—an idea supported since at least the late 1800s. Cardiac tissue and blood vessels were found to be widely innervated, as well as pericardial white adipose tissue (WAT; which is a very “brown” depot), in small rodents as early as the 1890s [1]. Direct evidence of innervation of brown adipose tissue (BAT; in mice and rats) was demonstrated in the mid-20th century [2]. Interestingly, even in the 1950s, Sidman and Fawcett made a statement that would be equally relevant today. They wrote, “Relatively little attention has been paid in recent years to the influence of the nervous system on adipose tissue even though detailed experimental studies on this subject are to be found in the (literature).” They went on to cite a number of studies in the 1930s that presented evidence for the importance of adipose nerves; and themselves stated that mixed nerve types innervate BAT and that many nerve fibers were not associated with blood vessels but extended into the parenchymal space and made direct contact with adipose cells [2]. Similarly timed investigations argued, with the support of fluorescent microscopy, that the sympathetic innervation found in BAT was restricted to the vasculature and was mainly vasoregulatory in function [3]; although, the existence of parenchymal nerve fibers in adipose tissue was at least becoming more accepted a year later [4]. 

Soon after, Bargmann et al. [5], in the late 1960s, used electron microscopy to demonstrate (in mice, rats, and hedgehogs) that not only did sympathetic fibers envelope BAT vasculature but that they also extended into the parenchymal space. Interestingly, as we describe below, this parenchymal innervation of adipose was rediscovered in recent years with whole-depot imaging techniques. Bargmann et al. [5] were also able to see both non-myelinated and myelinated paravascular (their anatomical term) nerve bundles, but more importantly, they found that small unmyelinated axons were tightly associated with fat cells themselves. Interestingly, the axon terminals of these nerves, some of which were “embedded in invaginations” of the adipocyte surface, contained synaptic vesicles that the authors presumed to contain catecholamines [5]. This presumption was based on the mounting evidence for catecholamine release driving adaptive thermogenesis in BAT in multiple mammalian species [6,7,8], findings which also underscored that NE was essential for this process instead of the previously hypothesized epinephrine/adrenaline [9,10]. 

Based on these studies, it became accepted that cold-induced thermogenesis caused an increase of NE release to BAT, which resulted in lipolysis and activation of uncoupling protein 1 (UCP1). However, if we look back to some of the original findings regarding adipose innervation [4], there may be another mechanism to consider. Early fluorescent microscopy imaging of BAT and WAT innervation argued for numerous nerve fibers surrounding the vasculature, predominantly around arteries and arterioles [3,4]. Given this perspective, an alternative hypothesis could be that cold-induced thermogenesis does not result in an increase in the concentration of circulating NE, nor might it result in an increase in direct sympathetic drive to WAT and synaptically released NE. Instead there could be stimulation of vasodilation to tissues due to vasoregulatory innervation and thus no resulting change in the absolute catecholamine levels delivered to the tissue per volume of blood, but rather an increased volume of blood that would thereby deliver more catecholamines. This idea could provide a vascular mechanism for how obese adipose tissues loses thermogenic capacity, given the vasculature damage that occurs due to chronic inflammation and the relative loss of vascular supply to adipocytes, due to expanding adipose mass coupled with a lack of new angiogenesis, as reviewed by Stapleton et al. [11].

These early microscopy studies could not benefit from the use of fluorescently-labeled antibodies targeting markers of sympathetic innervation, as they did not yet exist, thus instead they employed formaldehyde gas to form highly fluorescent isoquinoline derivatives, formed from the presence of monoamines (including NE) [4]. The resulting observations indicated the presence of neurotransmitters in axon terminals of BAT and WAT nerves, and may have actually been a more specific result than what we get with antibody staining today, given the potential for off-target and non-specific binding of the antibodies. The downside of this formaldehyde gas technique was the inability to distinguish the subtype of monoamine they were fluorescing.

### 2.2. Innervation of White Adipose Tissue (WAT)

The observation of Bargmann et al. [5] that each adipocyte comes in contact with nerve fibers in the parenchyma is consistent with what we and others have recently reported in WAT [12,13,14]. Earlier studies using True Blue as a retrograde neural tracer (crystal implants in WAT) showed sensory innervation in inguinal subcutaneous WAT (i-scWAT), as evidenced by tracing back to the T13/L1-L3 dorsal root ganglia (DRG) [15]. The anterograde transneuronal viral tract tracer, the H129 strain of the herpes simplex virus-1 (HSV-1), has also been used to trace sensory nerve projections from i-scWAT and intraperitoneal epididymal WAT (eWAT), through the same T13/L1 DRG in Siberian hamsters [16]. Together, these studies and others like them have indicated that adipose has bi-directional communication with the brain—through both afferent sensory fibers and efferent sympathetic fibers. However, the role of sensory innervation in WAT continued to be largely understudied in lieu of sympathetic nervous system (SNS) innervation studies in the intervening years. This made sense given the importance of NE for thermogenesis and lipolysis. More recently, new studies have revisited the function of adipose sensory nerves. Long-form leptin receptor (ObRb) was found on DRG neurons traced from i-scWAT, suggesting leptin could potentially communicate with sensory nerves in adipose via the DRG [17]. Furthermore, SNS-stimulated lipolysis, as well as intra-adipose injection of free fatty acids, such as eicosanoidpentaenoic acid (EPA) and arachidonic acid (AA), could increase adipose afferent nerve activity [18]. This was capable of triggering BAT thermogenesis, an effect that was abolished with surgical denervation of i-scWAT [18]. 

Although early microscopy studies provided evidence of WAT innervation and hypothesized that these nerves were of SNS origin [4], irrefutable proof of SNS innervation of WAT came from the seminal studies of Youngstrom and Bartness, who demonstrated bidirectional innervation of WAT [19]. Using the retrograde fluorescent tract tracer FluoroGold injected into i-scWAT and eWAT, as well as the anterograde fluorescent tract tracer DiI, they determined that the SNS ganglia also at T13/L1-L3 innervated both fat pads. These studies further showed that there was innervation of the adipocytes themselves, and not just the blood vessels within a depot, as had been previously demonstrated in the mid 1900s. The specificity of the tracing was confirmed by surgical denervation of the fat depots and by injecting the tracers directly into blood vessels. Furthermore, using pseudorabies virus (PRV) retrograde tracing from i-scWAT, which can only trace sympathetic neurons that are synaptically connected, they could create hierarchical connectome that mapped neuronal pathways from the tissue to the brain [20]. For the next two decades, the work of Timothy Bartness would continue to pioneer our understanding of adipose innervation in rodent models [21]. 

## 3. Lessons from Adipose Denervation Studies

Multiple methods for and consequences of denervation of WAT and BAT have been reported in the literature, and have been described in detail in recent reviews [22,23]. Of these, surgical denervation is considered the most effective at eliminating total neural input and output, as the nerve bundles innervating the tissue are physically resected. However, surgical denervation is generally non-specific to nerve type (as sensory and sympathetic nerve bundles tend to travel together), can be a technically challenging method, and can cause undesirable effects as nerve bundles that innervate adipose depots often innervate other tissues and can run along the vasculature. An alternative approach is chemical denervation. Chemical denervation can provide a selective and localized means of removing nerve supply to WAT depots. The first selective chemical denervation drug was 6-hydroxydopamine (6-OHDA) [24], which is taken up into NE storage vesicles leading to oxidative damage to the membrane and nerve degeneration, and thereby producing a reversible denervation of sympathetic nerves while leaving sensory nerves intact [22]. Later, some chemical denervation studies were undertaken using guanethidine to produce chemical sympathectomy. This drug works by displacing NE from postganglionic sympathetic nerve endings, and decreases reuptake of NE by nerve terminals. When locally administered, it can deplete a tissue of NE, effectively depleting sympathetic tone. It has been used to provide direct evidence of the role of NE in WAT, as local injection of guanethidine to i-scWAT resulted in increased fat pad size, due to adipocyte hyperplasia, in Siberian hamsters [25]. This denervation experiment helped provide a mechanism by which the SNS regulates body mass, which is to regulate adipocyte dynamics and cell size. The current preferred sympathetic denervation drug is 6-OHDA, as subsequent preparations of guanethidine proved less reliable [23]. 

For chemical denervation of sensory nerves, capsaicin is typically used, which activates vanilliod receptors on unmyelinated and myelinated sensory nerves. The over-activation of the receptors causes an influx of calcium and sodium resulting in an excitotoxic effect [22]. The efficacy of capsaicin-mediated sensory denervation is validated by a reduction in calcitonin gene-related peptide (CGRP) and Substance P content, and although it is not as effective as surgical denervation, this method does appear to leave sympathetic efferent nerves intact [26].

### 3.1. Denervation of BAT

Some of the most elegant studies demonstrating the importance of neural control for adipose tissue metabolism and function involved surgical denervation of BAT. By removing the neural supply to the organ, observed metabolic perturbations were directly linked to a lack of brain–adipose communication. Indirect evidence for sympathetic control of lipolysis had been demonstrated by studies showing an increased rate of NE turnover (NETO) in both BAT and WAT following cold exposure in rats [27]. Increased NETO in WAT has also been demonstrated with fasting in rats [28]. Denervation studies have added credence to these findings. Unilateral and bilateral denervation of interscapular (iBAT) is technically less difficult than denervation of WAT depots, as nerve bundles innervating iBAT are more easily visualized and anatomically defined (five intercostal nerves that unilaterally innervate iBAT) [29] than those innervating WAT depots [23], and originate mainly from the stellate ganglion [30]. Bilateral denervation of iBAT resulted in greatly impaired thermogenesis and reduced overall energy expenditure, increased body fat mass [31], and “whitening” of the tissue [32]. Furthermore, experiments with unilateral iBAT denervation showed decreased presence of tyrosine hydroxylase (TH) [32] and UCP1 [33] only in the denervated fat pad compared to the intact contralateral pad. In studies where sympathetic drive to BAT increased total energy expenditure, surgical denervation blunted the effect [32,34]. This surgical denervation of BAT was done by severing the large nerve bundles transiting to BAT, as anatomically defined [29], while leaving nearby vasculature intact [35]. Furthermore, bilateral chemical sympathectomy of iBAT increased NETO in i-scWAT [35], demonstrating adipose tissue crosstalk with the brain in the attempt to reestablish energy homeostasis. Chemical denervation of sensory innervation to iBAT also impaired thermogenesis [36], demonstrating the need for sensory feedback from iBAT for proper thermogenic function. 

### 3.2. Denervation of WAT

There are metabolically relevant consequences of losing the nerve supply to WAT. Surgical denervation of WAT led to increased fat pad mass and white adipocyte proliferation and differentiation, as demonstrated in both rats [37] and Siberian hamsters [19,38,39]. The i-scWAT fad pad is innervated by multiple nerve bundles entering the tissue at multiple locations. Surgical denervation of i-scWAT was accomplished by tracking these nerves under four-times magnification to their terminal location and bisecting the nerves at that area [19]. Retroperitoneal WAT (rWAT) is perhaps more easily surgically denervated by lifting the kidney and cutting the three nerve bundles right before they enter the fat pad [38]. These experiments underscored the importance of innervation for regulating hypertrophy versus hyperplasia, as well as for controlling levels of lipolysis. However, surgical denervation could not reveal which nerves are most essential in maintaining proper body mass and metabolic health, as the technique denervates both sympathetic and sensory nerves and causes disturbance of vasculature. 

The Bartness lab and others have subsequently used chemical denervation to gain a deeper understanding of which nerves act in adipose depots and how. Chemical symapathectomy (with 6OHDA) of one fat pad increased total body fat and increased adipocyte number in the contralateral fat pad in rats and mice [40]. One interpretation of these data would be that sensory feedback from the denervated pad led to effects in the non-denervated pad via the central nervous system. However, treatment with peripherally-administered leptin could reduce fat pad size independent of innervation status [40], confirming that endocrine and neuronal effects mediate adiposity. In subsequent studies, 6-OHDA denervation of sympathetic nerves in one or both fat depots reduced the NE content and inhibited NETO in other WAT pads as well as in iBAT [41]. 

Over and over, sympathetic denervation of WAT resulted in increased depot mass, characterized by an increase in cell number and a decrease in lipolysis, as recently reviewed [22]. On the other hand, sensory denervation of iWAT and eWAT increased fat pad mass via hypertrophy instead of hyperplasia, providing a means of differential control of WAT by sympathetic versus sensory nerves [26,39]. The next step in our understanding must be to delineate how different neuropeptides and neurotransmitters exert these effects, and whether sensory nerve products potentially modulate the effects of NE from SNS nerves.

### 3.3. Potential for Nerve-Independent Thermogenesis in Brown Adipocytes 

While the contribution of adipose innervation is clearly important for the regulation of metabolism and thermogenesis, and mice lacking beta-adrenergic receptors cannot easily survive the cold, several studies have attempted to clarify cell-autonomous effects that may circumvent neural inputs to regulate these processes. As is nicely reviewed in [42], beta-adrenergic signaling has differential effects on lipolysis, including activity of the enzymes hormone sensitive lipase (HSL) and adipose triglyceride lipase (ATGL). Non-adrenergic biomolecules can also impact lipolysis through actions on G-protein coupled receptors that act through similar intracellular pathways as adrenergic signaling (e.g., adenylyl cyclase, cAMP). Numerous of these have known neural functions as well, such as purine nucleosides (adenosine), melanocortins, glucocorticoids, and parathyroid hormone. Other receptor-independent pathways to lipolysis have also been uncovered, as well as alternate sources to produce intracellular free fatty acids as a means to activate UCP1 (also reviewed in [42]). What is less convincing; however, is the ability of brown adipocytes to directly sense cold temperature themselves. Cold stimulation has been well-described as stimulating TRP channels on skin sensory nerves, which send signals back to the brain, which leads to coordinated regulation of sympathetic outflow to stimulate thermogenesis. Cold-sensing mechanisms have not been described in a cell autonomous manner, although at least one study has reported a cold-stimulated in vitro culture condition was sufficient to turn on brown adipocyte genes, including UCP1, in cultured 3T3-F442A cells [43]. The underlying reason for this observation may have been cold stress instead of cold-induced thermogenesis, but the mechanism was not fully explored. Mice lacking the beta-adrenergic receptors were still able to stimulate a thermogenic program in scWAT, although the contribution of non-adrenergic nerve products was not explored as a potential mechanism [43].

## 4. Advancements in Imaging/Analysis Techniques for Visualizing Adipose Innervation

### 4.1. What Has Been Learned from Tissue Sectioning

To visualize and better understand the anatomy and physiology of adipose tissue, researchers have been processing adipose tissues in micron-thick sections for subsequent histological staining on slides or floating sections [3]. By this method, it is beneficial to stain for multilocularity, browning and UCP1 expression, or the presence of macrophages in crown-like structures. However, adipose innervation is not easily demonstrated in 7–10 µM thick sections because cross-sections of nerves appear mostly as puncta. This does not allow for the quantification of the total tissue innervation and also does not allow for adequate visualization of synapses. This is especially true as we now know that the innervation of WAT is heterogeneous in nature [12,13,14]. Given the significant interest in the innervation of adipose tissue that has recently re-emerged, it is; therefore, important to develop whole-tissue methods for visualizing and quantifying adipose innervation across a depot. The sympathetic innervation of BAT tissue has been well established due to its role in thermogenesis [44,45,46,47]. Conversely, WAT has been relatively understudied as a tissue of significant innervation, and these tools could help increase our knowledge. 

Several investigations have been conducted in the past 20 years that have used histological processing of thin-sectioned adipose tissue to elucidate the various nerve types residing there. Immunofluorescence imaging of thin tissue sections using TH as a marker for sympathetic nerve activity has been used extensively to document sympathetic innervation within both WAT [39,48,49,50] and BAT [51]. Tyrosine hydroxylase is the rate-limiting step in catecholamine synthesis and its expression goes up upon sympathetic nerve activation. Immunohistochemical staining for TH in subcutaneous and visceral adipose depots has been used to show an increase in tyrosine hydroxylase immunoreactive (TH^+^) SNS parenchymal nerve fibers after cold stimulation [51,52]. However, TH immunoreactivity is not a good method for assessing total innervation, as the levels of this enzyme fluctuate in response to SNS stimulation. 

It is well known that cold exposure can induce sympathetic drive in both WAT and BAT [53], accompanied by increased TH levels [54] and increased browning and UCP1 expression in WAT [55]. Interestingly, this drive was shown to be greater in females due to an increased expression of estrogen-dependent sympathetic nerves [56]. Warm temperatures elicit the opposite effect in BAT, thus decreasing sympathetic nerve activity and TH expression [57].

Sensory innervation has also been well established in adipose by marking afferent sensory nerves with their neuropeptide products, such as CGRP [39,50,58] and Substance P [50,58]. Both of these neuropeptides are associated with inflammation brought on by sensory nerve nociception [59,60], but it is still unclear what stimulates their secretion in WAT or BAT and how these might affect energy balance.

Studies conducted on rat BAT [61] have shown that only a small subset of brown adipose depots are parasympathetically innervated: pericardial BAT and mediastinal BAT [62]. There is; however, some uncertainty surrounding whether or not WAT is parasympathetically innervated. A study conducted in 2004 suggested the presence of parasympathetic nerves by using retrograde trans-neuronal tracer PRV to mark parasympathetic nerves in rats [63]. This was later refuted when WAT sections failed to be labeled by parasympathetic postganglionic nerve markers, including vesicular acetylcholine transporter (VAChT), vasoactive intestinal protein (VIP), and neuronal nitric oxide synthase (nNOS) in Siberian hamsters [64]. It was proposed by Berthoud et al. [65] that the significant vagal innervation findings were due to leaking of the retrograde tracer and improper controls, thus creating false positives. Kreier and Buijs [66] stated, in a letter to the editor, that the lack of VAChT, VIP, and nNOS staining is inadequate to rule out the significance of parasympathetic input in the WAT due to their highly variable nature within tissues known to have significant parasympathetic input, suggesting that there are no known universally-expressed parasympathetic markers that are equally expressed among all tissue types. They also questioned the ability of Giordano et al. [64] to perform a completely thorough chemical sympathectomy in WAT, stating that mechanical sympathectomy is required. This was soon followed by a rebuttal by Berthoud et al. [67] which argued against the possibility of a parasympathetically innervated tissue showing complete absence of all the markers in question. Giordano et al. [68] also rebutted Kreier and Buijis’s statements in a response aptly titled: *Reply to Kreier and Buijs: no sympathy for the claim of parasympathetic innervation of white adipose tissue.* In the decade since, little data have served to clear up the confusion. 

### 4.2. Whole-Tissue Processing and Imaging

The need for whole-tissue (or, whole-mount) imaging techniques became necessary as researchers wished to further their knowledge of neuronal interactions and the extent of synaptic connections within adipose tissues. Given the high lipid content in adipose and the brain, the autofluorescence of lipids was problematic in imaging these particular tissues in toto. Accordingly, a technique was needed to remove lipids from the tissue or blunt lipid autofluorescence. To chemically delipidate the tissue, methods were pursued to optically clear the tissues in order to reduce tissue autofluorescence and limit light adsorption, while having minimal effects on tissue morphology [69].

Since as early as 1911, clearing techniques have been implemented in various histological studies. Widely accepted to be the first clearing method published was a benzyl alcohol-methyl salicylate mixture used to aid in the visualization of anastomoses between coronary arteries in the heart [70]. Disappointingly, this method caused significant tissue deformity and damage due to excessive tissue shrinkage and superficial necrosis [71]. This clearing technique also lacked the crucial delipidation step that would be necessary for lipid-rich tissues such as the brain or adipose.

The first clearing method that included delipidation was a method originally developed for whole brains using benzyl alcohol/benzyl benzoate (BABB) [72]. This technique was slightly modified and used in the first published whole-tissue imaging study of adipose innervation conducted on mouse i-scWAT [12]. Several groups concurrently worked to develop similar protocols for adipose tissue whole-mount imaging [13,14]. 

Several other clearing techniques with delipidation have been applied to whole-adipose depots since then, in order to explore innervation. One such technique is iDISCO [73], which is another solvent-based clearing technique that has the added benefit of reducing the antibody fluorescence quenching that was problematic in traditional BABB clearing [74]. iDISCO is the basis for nearly all of the whole-depot clearing techniques currently published for adipose [14,75,76,77,78,79]. This being so, iDISCO, is far from an ideal clearing method. iDISCO has a fluorescent protein emission lifespan longer than that of many other clearing methods but it is still only a couple days long requiring immediate imaging of tissues [69,74]. iDISCO also does not preserve accurate tissue morphology due to tissue shrinkage and tissue hardening [74]. These factors should be a considered for any study that uses iDISCO as a clearing agent. 

The iDISCO clearing technique was further modified for use in adipose by the addition of a more thorough methanol/dichloromethane-based delipidation step. This adipose specific method has been termed Adipo-Clear [14,79]. Clearing techniques are continuing to evolve and have moved from clearing entire organs to clearing entire organisms [80,81] and will continue to be implemented in adipose-nerve studies as time moves on. Similar whole-tissue imaging techniques have even been applied to engineered adipose substitutes to allow for characterization of the vascular networks that reside in them [82].

The aforementioned clearing techniques as well as many others have been comprehensively reviewed for general tissue use [69,71,74] and for specific use in scWAT [13]. With the noted advancements in clearing techniques and whole-tissue 3D imaging of adipose tissue, it is important to note that clearing is not always necessary. Sudan Black B (which we now call Typogen Black) can be used to block a significant amount of lipid and lipofuscin autofluorescence. TrueBlack® Lipofuscin Autofluorescence Quencher should be used in place of Typogen Black when imaging in a far-red channel, due to its inherent fluorescence at longer wavelengths. Lipid/lipofuscin blocking should be followed by washes with 1X PBS with 10U/mL heparin. Heparin reduces the majority of autofluorescence from the vasculature. Tissue thickness in the z-direction can also be significantly reduced with only slight deformity to the tissue. This can be done by placing the tissue between two glass slides held together by large binder clips for 1.5 h at 4°C [13]. Many of the solvents used for tissue clearing, such as BABB or iDISCO, can be caustic to microscope lenses and the squishing technique stated above can avoid the need for purchasing special BABB safe lenses.

Since the interactions between nerves and vasculature are clearly important, techniques for visualizing adipose vascular supply have been developed, including the use of Isolectin-IB_4_ [77,83], which binds to group B erythrocytes, perivascular cells, and endothelial cells [84]. Isolectin-IB_4_ staining has the caveat of staining certain sensory nerves as well as vasculature [85], but stained nerves can be easily distinguished from stained vasculature due to morphology. Isolectin-IB_4_ staining in whole-mount adipose tissues has been used to show a decrease in adipose depot vascular supply in obese mice [77], and to investigate age-related neuropathy of the nerves surrounding vasculature in i-scWAT [13]. 

### 4.3. Discoveries Using Whole-Adipose Tissue Imaging

Many important discoveries have been made to increase our understanding of adipose nerve interactions that would have been impossible without the utilization of whole-tissue imaging techniques. The first study to publish observations on whole-mount adipose innervation did so by embedding a mouse i-scWAT depot in agarose, clearing it in BABB, and imaging the tissue with optical projection tomography before 3D reconstruction. This was the first time whole-depot images were taken that clearly showed vasculature and axon bundles in adipose, which were branching across the length of the tissue. In that same study they also showed adipose–nerve connections within an i-scWAT depot in vivo [12].

Two years later a study was published on i-scWAT innervation that used what the authors called a pan-neuronal marker; synaptophysin, the sympathetic marker TH, and the adipocyte marker perilipin [75]. Although often used as a pan-neuronal marker, synaptophysin is more accurately described as a pre-synaptic marker present on nearly all pre-synaptic vesicles and is limited by not being an actual axonal membrane protein [86,87].

The tissues from mice were optically cleared with a slightly modified iDISCO technique, imaged on a lightsheet microscope, and 3D reconstructed in a process they termed “volume fluorescence imaging.” Their findings showed that sympathetic nerve fibers were located in close contact with approximately 91.3% of all adipocytes, something that was first proposed in 1968 [5]. Synaptophysin and TH were 98.8% co-localized [75], which the authors suggested was evidence for the majority of i-scWAT nerves being sympathetic, leaving only 1.2% as a possible sensory and/or parasympathetic type. However, it is unclear whether this co-localization of synpatophysin and TH was observed under basal- or cold-stimulated conditions. Cold stimulation reversibly increases TH^+^ fiber density in i-scWAT [78] and would likely increase the number of presynaptic vesicles marked by synaptophysin. If these results are from cold stimulated animals then the data are possibly skewed and may be overestimating the ratio of sympathetic to sensory nerves in i-scWAT under basal conditions. This estimate also does not seem to fit with recently published images of sensory innervation of WAT [13], which demonstrate widespread sensory nerves (marked by Nav1.8) in i-scWAT. Additionally, immunofluorescence labeling of parasympathetic nerve marker ChAT revealed no more than 5 ChAT expressing nerves per i-scWAT depot [75], supporting earlier studies which found little to no parasympathetic fibers in WAT [64]. 

Further investigation into sympathetic innervation of WAT was conducted using the Adipo-Clear technique and lightsheet microscopy. Mice were cold exposed to elicit sympathetic activity and tissue browning. This study found significant intra-adipose variation in TH^+^ fibers between subcutaneous and visceral depots [14]. Tissue autofluorescence was used to show tissue morphology in this study [14]. 3D projections of sympathetic (TH^+^) nerves within both the i-scWAT and eWAT were reconstructed to show differences in arborization between tissue types. Chi et al. [14] described the sympathetic innervation of i-scWAT to be arborized into “discreet lobules”, whereas eWAT had an “amorphous structure”. It has since been revealed that the inguinal subcutaneous depot has a specific pattern of innervation. The largest nerve bundles, which we are now calling the subiliac transverse nerves, are located at the tissue center, which span the length of the tissue traversing across the subiliac lymphnode in conjunction with the thoracoepigastric vein. It is from these larger nerve bundles that the majority of the smaller nerves branch off from [13]. 

A number of similar studies have been conducted to investigate whole-adipose innervation with cold challenge. Whole-depot i-scWAT imaging and quantification of the pan-neuronal marker β3-Tubulin [13] and of TH^+^ nerve fibers in 0.3 mm^3^ representative sections [78] all suggest an increase in nerve arborization with cold challenge. It has also been shown that i-scWAT innervation returns to the room temperature state following rewarming, as indicated by a drastic reduction of neuronal arbors [13,78].

It is important to note that some of the studies using whole-mount tissue imaging of adipose [75,77,78] only quantify representative 3D-projection images of 0.3 mm^3^ sections for much of their findings, in lieu of the entire cleared depot. Due to the regional specific arborization patterns in i-scWAT, as previously described [13,14], it is apparent why whole-depot imaging and quantification of innervation is necessary and why representative sections cannot yield completely accurate results. 

## 5. Peripheral Nerve Regulation in the Pancreas, Liver, and Gut

Beyond adipose tissue, numerous peripheral metabolic organs have been used to demonstrate the importance of neural innervation and brain–adipose communication for the regulation of energy balance. Below we summarize recent findings from the pancreas, liver, and gut. Findings from these organs may inform the future study of adipose innervation and brain–adipose communication, as there may be similar mechanisms for regulation of peripheral innervation of these tissues.

### 5.1. Pancreas

The pancreas contains three primary secretory cell types, α-, β-, and δ-cells, that reside in the Islets of Langerhans and are responsible for the release of metabolic hormones such as glucagon, insulin, and somatostatin, respectively. Pancreatic islets are known to be highly innervated by the peripheral nervous system, particularly from fibers of vagal and celiac ganglion origin [88,89,90]. A discovery outlined in Paul Langerhans’ doctoral dissertation in 1869 [89,90] depicted a close relationship between hormone release and peripheral nerves, in this case non-myelinated nerves in rabbit and cat pancreas, with innervation observed in both the islets and the blood vessels proximal to the islets [89,90]. In recent research, humans have been shown to contain sparser innervation of the islets when compared to mice [91,92,93]. 

Neural regulation of secretory cells in the pancreas is supported by a number of studies. Acetylcholine, a neurotransmitter released by both sympathetic and parasympathetic nerves, is capable of stimulating hormone secretion in both β-cells and δ-cells [94,95,96]. α-cells are not known to be stimulated by acetylcholine [97] but do express the GABA_A_ receptor, which indicates that these cells may be regulated through inhibitory GABAergic signaling [98,99,100]. Interestingly, Ikegami et al. [101] have also uncovered increased GABAergic signaling in BAT of mice suffering from diet-induced obesity [101].

Diabetic polyneuropathy is a condition characterized by high levels of extracellular glucose, inflammation, and degradation/death of peripheral nerves, ultimately resulting in tissue necrosis due to lack of proper neural innervation and vascular supply. Neuropathic mechanisms are well documented in dermal layers but are also present in sub-dermal tissues including the pancreas [102], muscle [13,103,104,105], and scWAT [13]. The exact mechanisms mediating pancreatic neuropathy, or the reversal thereof, are not completely understood. However, current research in neuroimmune interactions aims to fill these gaps. 

Many studies have shown bi-directional communication between immune cells and peripheral nerves, indicating that neuroimmune interactions play a vital role in the progression of pancreatic diseases. Mast cells, known for their importance in allergy response, are also known to be involved in wound healing. In the pancreas, mast cells are speculated to contribute to neuroplasticity and pain severity by secreting nerve growth factor (NGF), which binds to TrkA receptors on sensory neurons and promotes the expression of Substance P [106,107]. This process is then exacerbated by NGF and Substance P-mediated mast cell recruitment and degranulation, respectively [107]. NGF is also known to be released by many other cell types including other immune cells [108], likely contributing to symptom severity. Demir et al. [109] have presented evidence of mast cells congregating perineurally in the rat pancreas, and Zhu et al. [110] have shown that anti-NGF treatment in a rat model of pancreatitis correlates with decreased nociception.

Interestingly, neurturin, a glial-derived neurotrophic factor, is increased in the human pancreas in chronic pancreatitis (CP), and is characterized by increased neural sprouting. DRG from rat were cultured in medium supplemented with human tissue extracts, prepared from patients with CP, and exhibited increased neurite outgrowth in cultured rat DRG cells when compared to controls [111]. This indicated a correlation between neural sprouting and neurturin levels in the pancreas. In addition, CGRP, Substance P, and Neuropeptide Y are known neuropeptides in the rat pancreas [112]. These peptides are major mediators of neuroimmune interactions in the periphery and are essential to the bidirectional communication seen between nerves and immune cells [108].

### 5.2. Liver

Hepatic innervation is similar to that of pancreatic innervation. In 1886 Walter Holbrook Gaskell reported, “An enormous number of non-medullated fibers stream out from these large ganglia to the intestines, stomach, liver, kidney…” [113]. These findings were further investigated by his scientific progeny F.H. Edgeworth, using the dog as a model animal; from which he determined that the nerve supply to the liver came primarily from the gastric plexus of the vagus, as well as sympathetic fibers from the celiac ganglia [114]. In 2006, Kreier et al. [115] investigated the interconnectivity of the nerves innervating the liver, pancreas, and adipose. Interestingly, rWAT was found to contain innervation of the same origin as the liver and pancreas, unlike i-scWAT, indicating differential innervation patterns for the two adipose depots and possibly preferential targeting of rWAT, which is known for easily browning, as a primary source of fuel [51]. 

Neural regulation of liver functions, such as glycogenolysis, the breakdown of glycogen secreted by α-cells in the pancreas into glucose, is variable among mammals. In guinea pigs and humans, sympathetic nerve fibers have been reported to extend intralobularly, ending deep in the parenchyma, while this was found to not be the case in liver of rats and mice [116,117,118]. It was suggested that a plethora of gap junctions among hepatocytes in these models may compensate for the lack of direct sympathetic stimulation [119,120,121]. Considering the speculative nature of these observations, more research is needed to uncover a definitive mechanism for how hepatic innervation affects hepatocyte and liver function. 

Regarding neural regulation of liver function, hepatic lipid metabolism was augmented with increased sympathetic drive. One study used a high-fat diet (HFD) supplemented with purified green tea catechins to stimulate sympathetic nerve activity, which led to decreased body weight and increased β-oxidation markers in the liver when compared to normal HFD mice [122]. Hepatic vagal innervation allows for bidirectional communication between the liver and brain, which is accomplished primarily through sensory and parasympathetic nerves. Localized disruption of vagal nerves innervating the liver with kilohertz frequency alternating current (KHFAC) [123,124] has exhibited beneficial metabolic effects in a clinical setting, including improved glycemic control in the human liver [125]. Interestingly, another study found that chemical denervation in the liver using 6-OHDA in rats resulted in impaired liver regeneration after injury but had no effect on liver function [126].

NGF is produced by a variety of cell types including immune cells, such as mast cells and macrophages [108]. In the liver, NGF is one of many signaling molecules used to promote a pro-inflammatory response when secreted by hepatic stellate cells (HSCs) [127]. The secretion of NGF via HSCs is interesting, since they comprise nearly 10% of the liver and are known to function primarily in the formation of scar tissue in response to hepatic injury. Kupffer cells are specialized resident macrophages of the liver that function as phagocytes to remove noxious substances delivered to the liver via the blood. These cells have been shown to express glial fibrillary acidic protein (GFAP), which is known as a mature astroglial marker in the central nervous system, as well as a glial-like marker in the periphery [128]. This again emphasizes the close relationship between glial cells in the brain and immune cells in the periphery. NGF is known to promote neuroplasticity in brain development [129] and in adult peripheral tissues [130]. Therefore, it is not unreasonable to believe that neurotrophic factor signaling plays a role in neural health throughout the liver, or in other peripheral tissues; however, more research must be done to elucidate specific mechanisms implicating NGF actions.

### 5.3. Gut

As may be expected from the proximity of the pancreas, liver, and gut, the origin of nerves innervating the gut is consistent with those innervating the pancreas and liver. Again, Gaskell in 1886 was a major contributor to the understanding of nerves innervating peripheral tissues, which included the gut [113,114]. One pivotal study that laid the groundwork for further research in this area was published by Hans-Rudolf Berthoud in 1991 [131]. In Berthoud’s study, dextran biotin, a bi-directional neuronal tracer, was used to trace nerves innervating the gut, resulting in a comprehensive map of vagal nerves. It was concluded that vagal innervation of the gut is primarily originated from the gastric branch, celiac branch, and a small contribution from the hepatic branch innervating the distal stomach [131].

Current research is investigating nerve sub-types and their roles in proper gut function. Cholecystokinin receptor (CCKR), a known afferent nerve receptor in the gut [132,133], was found to increase expression in the nodose ganglia (NG) of the vagus in response to obesity in high-fat diet fed diet-induced obesity prone (DIO-P) rats [134]. Recently, a method of selective ablation of afferent nerves (deafferentation) was developed, using a ribosomal inactivating protein, saporin (SAP), and CCK together as a conjugate. The SAP–CCK conjugate was unilaterally injected into the NG effectively blocking afferent nerve communication to the brain in rats, which was validated using immunostaining as well as behavioral assays [135]. This method is a useful tool for afferent denervation while sparing efferent fibers. Complementary to SAP–CCK ablation of vagal afferents, de Lartigue et al. [136] selectively deleted leptin receptors on NaV_1.8_ vagal afferent neurons. Genetic knockout of leptin receptors on sensory nerves in vagal afferents led to increased weight-gain, compared to wild-type controls, by preventing appropriate gut–brain signaling [136]. These studies provide essential evidence for the importance of gut–brain communication through vagal afferent innervation of the gut and their relationship to adipose tissue accumulation; while also complementing previous findings in adipose regarding the presence of the ObRb leptin receptor on DRG nerves innervating i-scWAT [17]. 

Bi-directional communication between gut resident nerves and immune cells has been an active area of study, and an interesting role for neurotrophic factors has been implicated in the progression and severity of inflammatory responses in the gut. NGF is secreted by a variety of immune cell types in the body including mast cells and macrophages, and promotes a plethora of signaling pathways including anti-inflammatory [108] and survival [108,137]. NGF signaling has also been linked to the expression of known sensory neuropeptides, CGRP and Substance P, in the rat gut [138]; however, the direct source of NGF secretion is not known due to the diversity of cell types capable of its production [108]. Interestingly, NGF also promotes the formation of colonic afferent central terminals (CACTs), which are localized to the dorsal horn of the spinal cord and increase visceral nociception in colitic mice [139]. 

### 5.4. Perspective

It is important to note that current research models using rodents to study the pancreas and liver indicate significant differences in the distribution of nerves and nerve types compared to that of humans [91,92,93,116,117,118], which may be problematic in the translation of this research to a clinical setting. Important distinctions have been outlined above regarding these innervation patterns. Although these models are limited in their translational power for human studies, the similarities in functional output of the organ or tissue could still be considered useful for further research of nerve–endocrine–immune interactions, as this cross-talk between physiological systems likely remains conserved. Interestingly, species differences were not as pronounced in models of gut innervation. Moreover, it is known that innervation patterns of the gut are conserved throughout a variety of species that span the breadth of the animal kingdom [140]. 

## 6. Adipose Neuroimmune Interactions

Although the role of immune cells, especially macrophages, in adipose tissue has been and continues to be an active area of study, neuroimmune interactions in adipose tissue remain largely obscure. It had been suggested that BAT and WAT macrophages synthesize catecholamines in response to cold [141]. Although Nguyen et al. argued that cold-induced adaptive thermogenesis requires alternatively activated (M2, anti-inflammatory) macrophages and did demonstrate that preventing this macrophage polarization resulted in an impaired thermogenic response, these findings were later challenged to suggest a different explanation for the observed relationship between macrophages and catecholamines. In 2017, Fischer et al. used a mouse model with genetic deletion of TH (the rate limiting enzyme in catecholamine production) in hematopoietic cells [142]. By this method, they refuted the findings that alternatively activated macrophages “synthesize” catecholamines. In their study, not only did deletion of TH in hematopoietic cells have no effect on energy expenditure, RNA sequencing on macrophages from various adipose tissues revealed that none of the macrophage populations tested contained transcripts for TH.

The debate between these two sets of findings may have found its resolution with some recent publications. Studies using zebrafish have demonstrated a role for macrophage signaling over long distances between non-immune cells during tissue developmental remodeling, whereby macrophages transported airineme vesicles between two different cell types [143]. Furthermore, buried in literature from the 1970s, was evidence that mouse peritoneal macrophages accumulated NE in vitro [144]. These studies allow for the possibility of macrophages transporting other materials, such as NE, within adipose tissues. 

Soon after, Pirzgalska et al. presented data that supported similar macrophage behavior but pertinent to neuroimmune interactions in adipose tissue [145]. They recently described a distinct macrophage population that associates in a specific manner with SNS nerves of i-scWAT. The appropriately named sympathetic neuron-associated macrophages (SAMs), can be found interacting with SNS fibers within WAT and are not only morphologically distinct from adipose tissue resident macrophages (ATMs), but exhibit a gene expression pattern distinct from adipose and other tissue macrophages [145], including expression of genes related to synaptic signaling, cell–cell adhesion, and neuron development. Unlike the circular morphology of ATMs, SAMs wrap around SNS fibers and exhibit an extended shape with long dendritic like projections. Like observations made by Nguyen et al., SAMs contained significant amounts of intracellular NE, but lacked the requisite enzymes for NE synthesis, as previously reported for macrophages [141,142]. These macrophages were of the Cx3cr1^+^ lineage and exhibited a pro-inflammatory state more similar to classically-activated than M2-type macrophages; however, their most distinguishing feature was the expression of solute carrier family 6 member 2 (Slc6a2; a known NE transporter), as well as monoamine oxidase A (MAOA), an enzyme that degrades NE. Although other macrophages have been shown to express MAOA [145], only SNS fiber-associating SAMs expressed Slc6a2 [145]. The authors proposed, quite believably, that SAMs were acting as an NE sink, taking up excess NE after SNS stimulation and degrading it. The transport of NE by SAMs was not explored. They also showed that SAMs are recruited to WAT in obesity (both diet-induced and genetic models) and may be contributing to the adipocyte hypertrophy through over-degradation of NE. They went on to show that ablation of Slc6a2 from SAMs in obese mice lead to an obesity rescue, through reestablishment of NE levels that served to increase lipolysis and energy expending processes, such as browning of WAT. SAMs (with analogous molecular machinery) were also identified in the SNS tissue of humans [145]. SAMs have been shown to associate with other neuronal tissues, such as the SCG and thoracic chains, and are also present in BAT [145]. However, their abundance in BAT is much lower than WAT, and their role in BAT may not be as metabolically relevant as it is in WAT, but this remains to be seen. 

In a parallel study, Camell et al. presented findings of ATMs in aged mice that degrade NE and contribute to age-related lipolysis impairment in visceral adipose tissue (VAT) [146]. ATMs from 24-month-old mice showed increased expression of MAOA and other catecholamine degrading enzymes in a NOD-, LRR-, and pyrin domain-containing (NLRP)3 inflammasome dependent manner, compared to young three-month-old mice [146]. Furthermore, Camell et al. independently showed that certain ATMs were closely associated with TH^+^ nerves in VAT [146]; providing the likelihood that what they called nerve-associated macrophages (NAMs) may be the same cells as SAMs. Although Camell et al. used a LysM-Cre:mTmG reporter mouse model to visualize their ATMs, which is a broader myeloid marker than the Cx3cr1^+^ model Pirzgalska et al. used, they did not investigate whether NAMs expressed Slc6a2 (SAM marker) even though the morphology of their NAMs is consistent with that of SAMs. 

Mutations in another type of Cx3cr1^+^ macrophage that do not appear to be SAMs have been linked to decreased BAT innervation and subsequent loss of homeostatic energy expenditure [147]. Mice with Mecp2 (methyl-CpG-binding protein 2) deficiency in a subset of BAT-resident Cx3cr1^+^ macrophages, exhibited lower expression of UCP1, a paucity of BAT tissue, and developed obesity after three to four months of age [147]. Interestingly these findings were BAT-specific and no changes were observed in WAT. Furthermore, tamoxifen inducible *Cx3cr1^cre^:Mecp2^fl/y^* mice were created to restrict Mecp2 deficiency from macrophage precursor cells (including brain microglia), and which also allowed for the mutation to be induced at a stage when tissue macrophages were mature. These animals were shown to respond to acute cold challenge, which appeared to rescue the BAT impairment [147]. Mecp2 is an ubiquitously-expressed nuclear transcription regulator [148] that maintains mature neurons and synaptic connectivity [149]. Mecp2 deficient macrophages showed an up-regulation of PlexinA4, which is known to signal through semaphorins to guide axonal growth [150,151]. Wolf et al.; thus, argued that overexpression of PlexinA4 in Mecp2 deficient macrophages inhibits axonal outgrowth in BAT, thus diminishing its function and ability to maintain homeostatic thermogenesis. They did confirm the presence of Sema6A^+^ neurons in BAT tissue, which supports their working model, as PlexinA4 reverse-signals through Sema6a to inhibit axonal outgrowth [151]. However, the question remains as to what overcomes this axonal inhibition with cold exposure, since acute cold exposure appeared to rescue the impaired phenotype. Cold exposure increases NE content in adipose tissue. Macrophages along with other immune cells express adrenergic receptors that bind NE [152], and have been shown to produce neurotrophic factors such as NGF [153] and brain derived neurotrophic factor (BDNF) [154] in human peripheral blood and the human brain [154], suggesting another possible mechanism in regulation of axonal plasticity may exist in adipose tissue, whereby immune cells are synaptically wired and release neurotrophic factor to nearby nerves.

In most studies mentioned above, it was clear that Cx3cr1^+^ immune cells are implicated in the neuro–immune interaction in adipose tissue. These findings suggest that multiple subsets of Cx3cr1^+^ macrophages work in concert to maintain energy homeostasis though interactions with the neural innervation of adipose depots. There are many more studies necessary to fully understand the diversity of these immune cells, and what neuroimmune role Cx3cr1^−^ macrophages may play in adipose tissue, if any. One issue that may have been addressed by the data described above is that definitions such as “classically activated” or “alternatively activated” (i.e., M1 or M2) macrophages are not sufficient labels when describing the diverse populations of macrophages in adipose tissue. This has been well-understood by immunologists for many years, and the adipose field is now catching up to this idea.

## 7. Perspective

**Figure 1:**
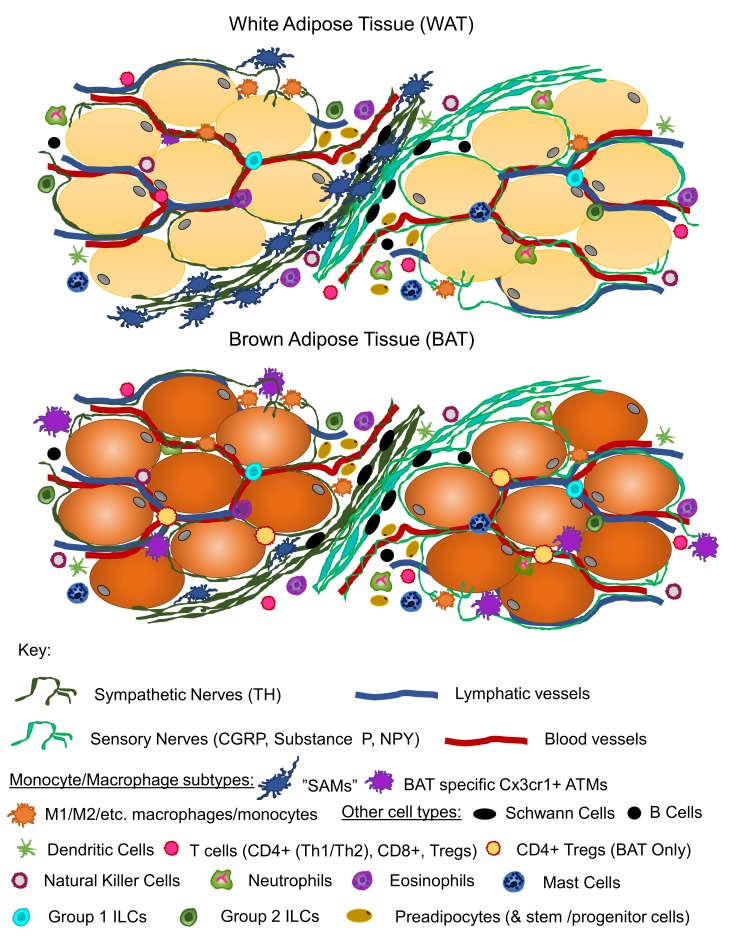
Architecture and neuroimmune cell types in white (WAT) and brown adipose tissue (BAT) in the basal state. Both WAT and BAT are comprised of lipid-laden mature adipocytes, where white adipocytes have one large (or unilocular) lipid droplet and brown adipocytes have many small (or multilocular) lipid droplets. The stromal vascular fraction (SVF) is the non-adipocyte cell fraction of the tissue, and contains preadipocytes (and stem/progenitor cells that will undergo white or brown adipogenesis) and a milieu of immune cells. Immune cell populations include innate immune cells, such as several subtypes of monocyte-macrophages, dendritic cells, mast cells, neutrophils, eosinophils, and innate lymphatic cells (ILCs); as well as adaptive immune cells, such as several subsets of T cells, natural killer cells, and B cells. Neurovasculature of WAT and BAT includes blood vessels, lymphatic vessels, and a dense nerve supply of both sensory and sympathetic fiber types, although it is currently unclear if one tissue has a greater extent of innervation than another, or if their nerve plasticity (such as with cold or exercise) differs between tissues or depots. Some nerves innervate the vasculature itself, while other nerves innervate the parenchyma of the tissue. It is currently unclear which cell types are directly innervated and receive synaptic input. Some nerve fibers are myelinated and others are unmyelinated (it has been suggested that the majority of TH^+^ nerve fibers in BAT are thin and unmyelinated [155]), and sensory nerve products such as calcitonin gene-related peptide (CGRP), Substance P, and neuropeptide Y (NPY) [26,50,155,156] have been detected in both tissues. Although, NPY may also be released from vasculature-associated sympathetic nerves as well [157], and should not be considered, by itself, a marker of sensory innervation. Sympathetic nerves release NE. Additional neurotransmitters and neuropeptides may also be active in WAT and BAT. The presence of Schwann cells, in both BAT and WAT, further underscores the presence of myelinated nerves [158]. Interestingly, Schwann cells, as a myelinated glial cell type, can behave similarly to an immune cell. Other immune cells, that are also present in adipose tissue, are well documented for neuroimmune functions, including monocyte/macrophage subtypes, as reviewed previously [159,160]. Monocytes, as well as T and B cells can produce BDNF in human peripheral blood and in human inflammatory brain lesions [154]. Human CD4^+^ T cell clones (from peripheral blood) were shown to produce and release NGF in vitro [153]. In addition, eosinophils from human peripheral blood can produce nerve growth factor (NGF), neurotrophin-3 (NT3), and brain derived neurotrophic factor (BDNF) neurotrophic factors upon immunologic stimulus [161,162], and mast cells contain mRNA for NGF and may be another source of neurotrophic factor [163]. It is currently unclear if these immune cell types play similar roles in adipose tissue, or if other tissue-resident immune cells have neuroimmune roles. Differences between BAT and WAT immune cells include more SAMs in white than brown [145], the presence of BAT specific Cx3cr1^+^ macrophages that play a role in regulating axonal outgrowth [147], and the presence of a subset of T_regs_ (CD4^+^) that exhibited a differential gene expression in BAT versus visceral adipose tissue [164]. There is very little knowledge about the lymphatic system in BAT compared to WAT, and changes with obesity or cold likely differs between BAT and WAT as well, but this warrants further exploration. In total, cellular cross-talk in the adipose organ is clearly important for immunometabolic function and brain adipose communication, but our understanding is still at a very primitive stage.

In summary, we are in the midst of an exciting time for the investigation of adipose tissue innervation and brain–adipose neural communication, given the flurry of recent publications providing new insights into innervation patterns, neuroimmune interactions, and regulation of adipose neuropathy and nerve plasticity in adipose depots (Figure 1). In addition, the new research tools that are being developed to genetically ablate nerves from specific tissues, including perhaps distinct adipose depots, as well as new microscopy methods for whole-depot imaging of nerves, will further provide new knowledge regarding how nerve supply is regulated in peripheral metabolic tissues in order to regulate whole-body energy balance and metabolic health.

## 8. Open Questions in the Field

1. Which nerve subtypes (including sensory vs. sympathetic and myelinated vs. unmyelinated) are present in BAT vs. WAT (and distinct depots thereof) and are these nerve types “plastic” (i.e., undergo remodeling in response to physiological or pathophysiological stimuli).
2. Are similar mechanisms of neural regulation and brain cross-talk conserved between different metabolic tissues and organs?
3. What is the role of tissue-resident immune cells in mediating nerve health, function, and plasticity?
4. What is the functional significance of the heterogeneity of nerve distribution across an adipose depot and which cell types have synaptic connections?
